# Phenotypic Modifications in *Staphylococcus aureus* Cells Exposed to High Concentrations of Vancomycin and Teicoplanin

**DOI:** 10.3389/fmicb.2016.00013

**Published:** 2016-01-25

**Authors:** Fábio D. A. Gonçalves, Carla C. C. R. de Carvalho

**Affiliations:** iBB–Institute for Bioengineering and Biosciences, Department of Bioengineering, Instituto Superior Técnico, Universidade de LisboaLisbon, Portugal

**Keywords:** *Staphylococcus aureus*, persistent infection, viable but non-culturable cells, stress response, vancomycin, teicoplanin

## Abstract

Bacterial cells are known to change the fatty acid (FA) composition of the phospholipids as a phenotypic response to environmental conditions and to the presence of toxic compounds such as antibiotics. In the present study, *Staphylococcus aureus* cells collected during the exponential growth phase were challenged with 50 and 100 mg/L of vancomycin and teicoplanin, which are concentrations high enough to kill the large majority of the cell population. Colony-forming unit counts showed biphasic killing kinetics, typical for persister cell enrichment, in both antibiotics and concentrations tested. However, fluorescence microscopy showed the existence of viable but non-culturable (VBNC) cells in a larger number than that of possible persister cells. The analysis of the FA composition of the cells showed that, following antibiotic exposure up to 6 h, the survivor cells have an increased percentage of saturated FAs, a significant reduced percentage of branched FAs and an increased *iso*/*anteiso* branched FA ratio when compared to cells exhibiting a regular phenotype. This should result in lower membrane fluidity. However, cells exposed for 8–24 h presented an increased branched/saturated and lower *iso*/*anteiso* branched FA ratios, and thus increased membrane fluidity. Furthermore, the phenotypic changes were transmitted to daughter cells grown in drug-free media. The fact that VBNC cells presented nearly the same FA composition as those obtained after cell growth in drug-free media, which could only be the result of growth of persister cells, suggest that VBNC and persister phenotypes share the same type of response to antibiotics at the lipid level.

## Introduction

Particular attention has been given to a fraction of bacterial cells which manage to remain alive even when the susceptible majority of biofilm-contained cells and/or planktonically grown cells are killed (Lewis, 2001, 2007). In the presence of antibiotic concentrations much higher than the minimum inhibitory concentration (MIC), cells of this subpopulation are able to survive while their counterparts are killed. These cells are now known as “persister cells” and were first described by Joseph W. Bigger who studied the action of penicillin against staphylococci (Bigger, 1944).

Contrarily to antibiotic-resistant bacteria such as VRSA or MRSA, persister cells are phenotypic variants belonging to a subpopulation of normal cells. Persister cells are responsible for the recalcitrance of chronic infections because they arrest growth or grow slowly and do not die in the presence of antimicrobial agents (Balaban et al., 2004; Dawson et al., 2011). When exposed to such stress, bacterial populations follow a biphasic killing curve, with a rapid killing phase followed by a distinct plateau of surviving persisters (Balaban et al., 2004; Patra and Klumpp, 2013). According to Lechner et al. (2012), persister tolerance is not transferred to the progeny. In fact, persister cells are genetically identical to their non-tolerant counterparts, suggesting that this strategy to survive a temporary environmental stress is based on phenotypic modifications (Dawson et al., 2011; Patra and Klumpp, 2013). The challenges in identifying persister genes have been numerous, and no mutants completely lacking the persister phenotype have been obtained, thus suggesting the existence of redundant mechanisms of persister formation (Hansen et al., 2008; de Groote et al., 2009).

It has been thought that persister cells are dormant variants of regular cells (Lewis, 2007, 2010). However, several studies showed recently evidence of an actual metabolically active character of persister cells (Hofsteenge et al., 2013; Johnson and Levin, 2013; Orman and Brynildsen, 2013a,b; Wakamoto et al., 2013). Nevertheless, before persister-related (chronic) infections may be eradicated, detailed knowledge regarding this strategy of bacterial adaptation is necessary. In particular, information addressing how persisters are formed and what really distinguish them from non-tolerant cells is of paramount importance.

*Staphylococcus aureus*, a Gram-positive bacterium, is one of the best-studied biofilm-producing organisms (Lewis, 2007) being implicated in cystic fibrosis and chronic lung infections (Goss and Muhlebach, 2011), and device/implant-related infections (Costerton et al., 1999; Singh et al., 2009; Grant and Hung, 2013), but the biofilms do not have increased resistance when compared with planktonic cells (Lewis, 2007). In the present study, we’ve exposed planktonic populations of *S. aureus* to concentrations of antibiotics several fold higher than the MIC to determine the phenotypic modifications occurring in the survivor cells, namely changes in the fatty acid (FA) composition of the phospholipids of the cellular membrane and of the cell surface properties. The method used, based on previously described techniques to produce persister samples (Keren et al., 2004; Hsu et al., 2011; Cañas-Duarte et al., 2014), consists in promoting the lysis of normally growing cells and sedimenting the remaining cells by centrifugation. However, by using fluorescence microscopy to assess cell viability, it was found that the survivor subpopulation contained more viable but non-culturable (VBNC) cells than persisters. Recent studies (Li et al., 2014; Ayrapetyan et al., 2015b) have defended that persister and VBNC cells coexist, are induced by the same conditions and may be part of a shared “dormancy continuum.” Nevertheless, all authors agree that the main distinction between persisters and VBNC cells is the ability of the former to resume normal growth when placed in fresh drug-free medium. Current isolation techniques do not allow the separation of persister and VBNC cells and most of the studies referring to persister cells must have been actually studying mainly VBNC cells (Orman and Brynildsen, 2013b). Since both types are in fact cells that have been able to maintain membrane integrity when their normal counterparts have suffered cell lysis, this indicates that if they are not the same subpopulation at least they must share similar adaptive mechanisms.

Alterations of the FA composition in bacteria are known to be an adaptive mechanism during environmental challenges such as changes in pH, temperature and osmotic pressure, as well as in the presence of toxic compounds (Zhang and Rock, 2008; de Carvalho et al., 2009; de Carvalho, 2012). Phenotypic changes related to the membrane, including increased fluidity and increased positive surface charge associated with FA content and phospholipid composition, have been observed in *S. aureus* exposed to daptomycin (Jones et al., 2008) and antimicrobial peptides (Nawrocki et al., 2014). Besides, the study of Mirani and Jamil (2013) showed that vancomycin has a significant impact on the phospholipid composition of vancomycin-resistant, -intermediate, and -susceptible *S. aureus*: the cells presented a high rate of conversion of saturated to unsaturated FAs and vancomycin-resistant and -intermediate strains presented no short-chain FAs. It was thus expected that the phenotypic variants, able to survive antibiotic concentrations much higher than the MIC, of the susceptible *S. aureus* strain used in this study also used modifications in the FA composition as an adaptation mechanism. To our knowledge, this is the first work studying lipid modifications occurring in cells challenged to very high concentrations of vancomycin and teicoplanin. The prospective role of membrane composition in the survival of these cells could help in the development of effective and specific therapies for persister-related infections.

## Materials and Methods

### Bacterial Strain

Strain *S. aureus* ATCC 25923 was used in this study. This strain is usually used as a vancomycin-susceptible control strain in studies comparing tolerance/resistance of *S. aureus* isolates. Bacterial growth was carried out in Mueller-Hinton broth (MHB; Fluka Analytical, Sigma-Aldrich) at 37°C and 200 rpm in an Agitorb 200 incubator (Aralab), unless stated otherwise.

### Determination of the Minimum Inhibitory Concentration (MIC)

The MICs for vancomycin (vancomycin hydrochloride from *Streptomyces orientalis* with a potency ≥ 900 μg/mg from Sigma-Aldrich, St. Louis, MO, USA) and teicoplanin (from *Actinoplanes teichomyceticus* with a purity ≥ 80% purchased from Sigma-Aldrich) were determined according to the Clinical and Laboratory Standards Institute (CLSI) guidelines (CLSI, 2014). In summary, the antibiotics were serially diluted in twofold steps (from 100 to 0.037 mg/L) in 96-well microplates (Sarstedt, Inc., Newton, MA, USA) in MHB. To 150 μL of medium containing the antibiotic in each well, 50 μL of an exponentially growing cell culture diluted to 0.5 McFarland standard was added. The microplates containing *S. aureus* cells were incubated at 37°C. The MIC was determined for each antibiotic, after 16 h of exposure, by visual inspection and by measuring the optical density of cell cultures in a SpectraMax^®^ Plus 384 Microplate Reader spectrophotometer (Molecular Devices, Silicon Valley, CA, USA) at 600 nm. At least two independent assays were performed. The MIC was defined as the lowest antibiotic concentration able to inhibit visible bacteria growth after 16 h of incubation at 37°C.

### Time-Dependent Killing

To 1 mL of *S. aureus* exponentially growing cells in 12 mL pyrex tubes, vancomycin or teicoplanin were added to reach a concentration of 50 or 100 mg/L. Since these concentrations represent over 100-fold MIC, we presumed that this treatment would result in the characteristic biphasic-killing curve, representative of persister formation, where the initial rapid killing of the majority of the population is followed by a plateau of drug-tolerant survivors. The pyrex tubes were incubated on a shaker at 200 rpm and 37°C for a total of 24 h. At certain time intervals (30 min, 1, 2, 3, 4, 5, 6, 8, 16, and 24 h), a tube was taken from the incubator, the cells were collected by centrifugation (9,680 *g* for 5 min), were washed and re-suspended in 1 mL of fresh MHB containing no antibiotic. From this cell suspension, 20 μL were sampled and spread on a glucose-yeast extract agar plate which was incubated at 37°C for determination of colony-forming units (CFUs). The CFUs were counted after 16–18 h according to CLSI guidelines (CLSI, 2014) or after 37–38 h of incubation when growth was slow (following the longest antibiotic exposure times). The assays were performed in triplicate.

### Growth After Antibiotic Exposure

*Staphylococcus aureus* cultures were challenged with 50 and 100 mg/L vancomycin or teicoplanin as stated in Section “Time-Dependent Killing.” After 3, 4, 5, 6, 8, 16 and 24 h, the 1 mL of culture medium was collected, the cells were centrifuged (9,680 *g* for 5 min), were washed and re-suspended in 1 mL of fresh MHB containing no antibiotic. This cell suspension was added to 19 mL of fresh MHB in 100 mL Erlenmeyers. Growth was promoted at 37°C and 200 rpm and monitored by measuring the optical density of the media at 600 nm until stationary phase was reached. The assays were done in duplicate.

### Cell Viability Analysis

Cell viability was assessed, during exposure to 50 and 100 mg/L vancomycin or teicoplanin, by fluorescence microscopy using an Olympus CX40 microscope equipped with an Olympus U-RFL-T burner and an U-MWB mirror cube unit (excitation filter: BP450–480; barrier filter: BA515). Cells were stained using a LIVE/DEAD^®^ *Bac*Light^TM^ Bacterial Viability Kit (Molecular Probes; Life Technologies; Thermo Fisher Scientific) and images were captured by an Evolution^TM^MP5.1 CCD color camera using the software Image-Pro Plus (both from Media Cybernetics, Inc., USA). The kit contains a mixture of SYTO^®^9 which stains all bacteria green and propidium iodide which stains red only bacteria with damaged membranes since it reduces Syto^®^9 fluorescence when both dyes are present. Image analysis was performed as described previously (de Carvalho et al., 2003). At least 15 images were taken from each sample.

### Lipid Analysis

To assess the FA composition of the cells, 1 mL of cell suspension was collected from each culture (following each exposure time and also cultures in drug-free media) and the cells were recovered by centrifugation at 10,000 *g* for 5 min in 1.5 mL eppendorf tubes (Eppendorf, Hamburg, Germany). Cells were washed twice with 1 mL of milli-Q water. The FAs from the cells were simultaneously extracted and methylated using the Instant FAME^TM^ procedure from MIDI, Inc. (Newark, DE, USA). The resulting fatty acid methyl esters (FAMEs) were analyzed by gas chromatography on a 6890N gas chromatograph from Agilent Technologies (Palo Alto, CA, USA), with a flame ionization detector (FID) and a 7683 B series injector and equipped with a 25 m long Agilent J&W Ultra 2 capillary column, also from Agilent. The gas chromatograph was programmed and controlled by the Sherlock software package, version 6.2, from MIDI, Inc. The FAMEs were identified by the software, using MIDI calibration standards, a methyl *cis*-11-octadecenoate standard solution from Sigma-Aldrich and confirmed by using two qualitative standards, one containing a mixture of bacterial FAMEs (Bacterial Acid Methyl Ester, BAME Mix) and another of polyunsaturated FAs (PUFA No.3 from menhaden oil), both from Supelco (Sigma-Aldrich).

### Zeta Potential Analysis

The electrophoretic mobility of the cells was determined using a Zetasizer Nano ZS from Malvern Instruments Ltd. (Malvern, Worcestershire, UK), as previously mentioned (de Carvalho, 2012). For that, 1 mL of cell suspension was collected from each culture, the cells were washed twice using milli-Q water, and resuspended in 1 mL of fresh milli-Q water. From this suspension, 20, 40, or 60 μL were collected and added to 2 mL of a 10 mM potassium nitrate (KNO_3_) solution in 2 mL eppendorf tubes. The different amounts ensured that sufficient cells were present for the analysis in case the antibiotics had caused more or less cell death than expected. The zeta potential, which is an indirect measure of the cell surface charge, was determined from the electrophoretic mobility according to the method of Helmholtz–von Smoluchowski by the software Zetasizer 7.10 from Malvern. The assays were done in triplicate and the results presented are average values.

### Bacterial Respiratory Activity Analysis

*Staphylococcus aureus* cell cultures were exposed to the certain antibiotic concentrations and the oxygen consumption rates were compared to that of non-exposed cells. The assays were performed on Oxodish^®^ OD24 microtiter plates, using a SDR Sensor Dish^®^ Reader (from PreSens Precision Sensing GmbH, Regensburg, Germany) to monitor the dissolved oxygen concentration. Data acquisition was carried out by the software SDR_v37 also from PreSens. The Oxodish^®^ microtiter plates have a resolution of ±0.4% O_2_ and a precision of ±1% O_2_ at 20.9% O_2_ and a drift <0.2% O_2_ within 1 week, according to the manufacturer. To test the effect of antibiotic concentration, cell cultures with an initial concentration equivalent to 0.5 McFarland standard were exposed to vancomycin or teicoplanin at concentrations from 2 to 100 mg/L. The plates were incubated at 37°C and 200 rpm and growth was followed by the dissolved oxygen concentration, as previously showed (Marques et al., 2012). Wells containing only medium and medium with 50 mM sodium sulphite were used as control for minimum and maximum oxygen consumption, respectively. Sodium sulphite is an oxygen scavenger since it reacts with available oxygen, producing sodium sulfate. The assays were done in duplicate.

### Assessing Enzymatic Activity

Semiquantification of the enzymatic activities of *S. aureus* cells before and after exposure to antibiotics, and after subsequent growth in fresh MHB containing no drug, was assessed using an API^®^ZYM kit from BioMérieux (France) according to the manufacturer’s instructions. Briefly, the cells were diluted in API suspension medium and added to each cupule. A surface-active agent (ZYM A) and ZYM B reagent were added and the mixture was left for at least 5 min for color development. A semiquantitative scale was used to classify color intensity: 0 (no activity) to 5 (strong activity). The assays were done in duplicate. A quality control of the API^®^ ZYM kit was carried with the enzymes β-glucosidase and α-chymotrypsin (both from Sigma), as suggested by the manufacturer. The API^®^ZYM kit has been used for the characterization and identification of bacteria (Bascomb and Manafi, 1998).

### Statistical Analysis

A Student’s *t*-distribution test quoted to a significance level of *p* < 0.05 was used to determine if the number, FA composition and zeta potential of persister cells were significantly different from normally growing cells under the different antibiotic concentrations tested.

Unsupervised principal component analysis (PCA) of the FA composition of the cells was performed with MetaboAnalyst 3.0 (www.metaboanalyst.ca; Xia et al., 2015), after auto-scaling of the data (mean-centered and divided by the standard deviation of each variable). PCA was applied to FA data to study the proximity of the cells cultured under the different conditions tested. This method indicates (indirect) gradients by producing a smaller set of variables, called principal components, able to explain the variability of a larger set of data.

## Results

To induce dormancy and assess the phenotypic changes, at the cellular membrane level, the *S. aureus* cultures were exposed to concentrations of vancomycin and teicoplanin much higher than the MIC. The lipid composition of the cells was analyzed and compared to that of non-exposed cells. We’ve also compared the oxygen uptake rate of the population during exposure to different vancomycin and teicoplanin concentrations and analyzed the enzymatic profile of the cells to infer on their metabolic state. The several stages of the study are described in the three sections below.

### Inducing Persister Phenotype with Vancomycin and Teicoplanin

To study the lipids of both regular and persister cells, the first step was the induction of the persister phenotype in *S. aureus* with 50 and 100 mg/L of vancomycin and teicoplanin. The MICs for vancomycin and teicoplanin, under the conditions tested, were 0.59 and 0.39 mg/L, respectively (data not shown). The vancomycin and teicoplanin concentrations used to induce persister phenotype are higher than the recommended therapeutic doses. For comparison, in adults with normal renal function, the usual daily dose of vancomycin is 2 g divided into doses given at 6 or 12 h intervals, which results in peak concentrations of 30–35 mg/L with trough levels of 5–10 mg/L (Stein and Wells, 2010). For life-threatening infections such as bacteraemia, endocarditis, meningitis and hospital-acquired pneumonia caused by *S. aureus*, trough serum concentrations of 15–20 mg/L are recommended (Rybak et al., 2009). A direct relation between nephrotoxicity and vancomycin serum concentration is still unclear, with toxicity being also dependent on other factors such as patient condition and duration of the treatment (Rybak et al., 2009; Gupta et al., 2011). Pre-dose serum concentrations of teicoplanin lower than 20 mg/L are usually considered appropriate for treatment of bacteraemia caused by MRSA (Darley and MacGowan, 2004).

It has been suggested that antibiotic concentrations much higher than the MIC kill rapidly the majority of the bacterial population, allowing the isolation of the persister population (Dhar and McKinney, 2007; Lechner et al., 2012). The resulting population killing curve will thus present a biphasic curve, with a fast killing phase corresponding to the majority of the dying population and a slowly decreasing phase representing the surviving persister cells (Balaban et al., 2004; Lewis, 2007).

**FIGURE 1:**
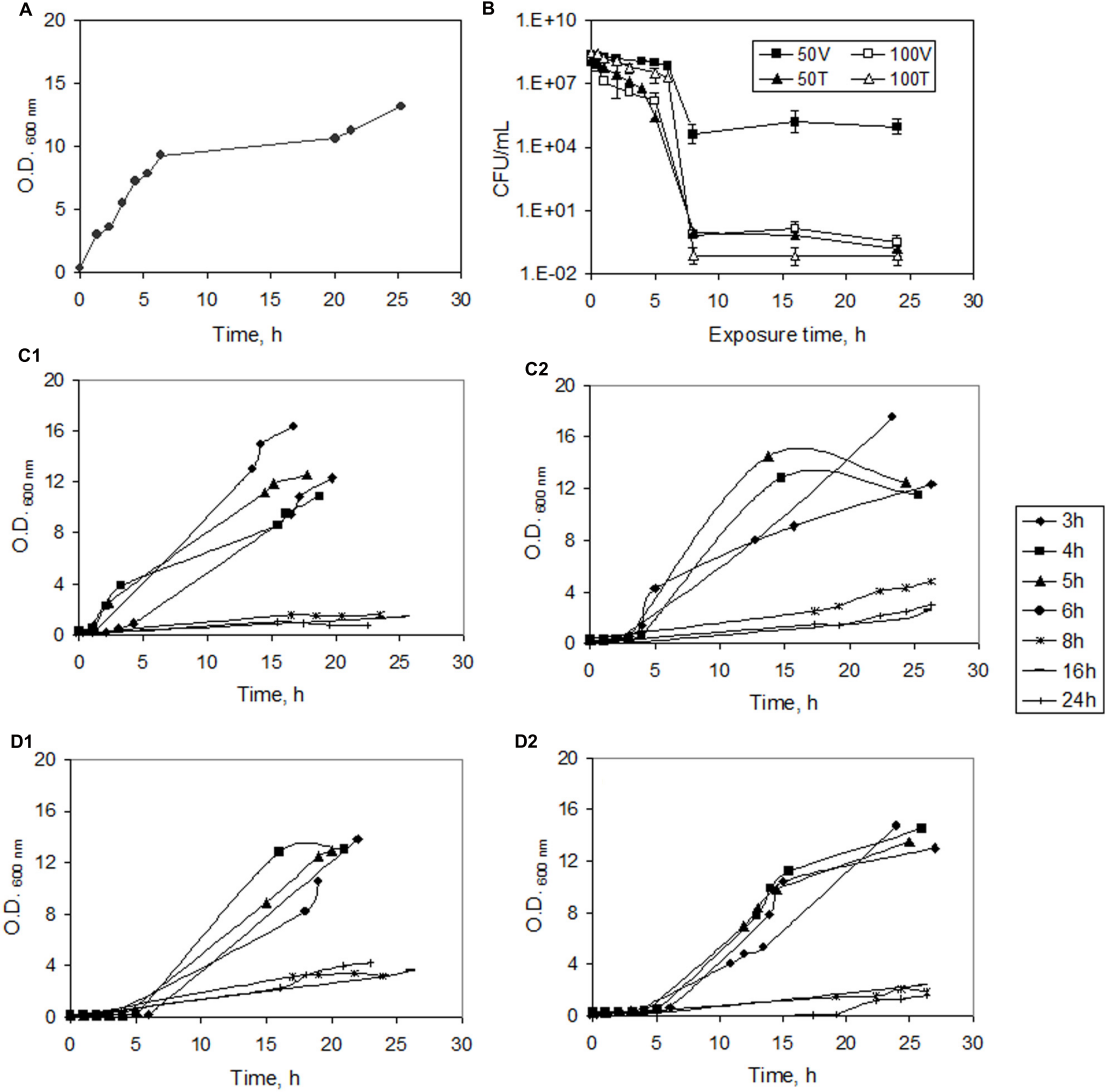
**Inducing persister formation in *Staphylococcus aureus*. (A)** Growth curve of normal cells in MHB. **(B)** Time-dependent killing curve during exposure to 50 and 100 mg/L of vancomycin (V) and teicoplanin (T). The CFU/mL data are represented in logarithm scale. **(C)** Growth curves of *S. aureus* in fresh MHB after exposure to 50 (C1) and 100 (C2) mg/L vancomycin for 3, 4, 5, 6, 8, 16, and 24 h. **(D)** Growth curves of *S. aureus* in fresh MHB after exposure to 50 (D1) and 100 (D2) mg/L teicoplanin for 3, 4, 5 and 6, 8, 16, and 24 h. Error bars represent standard deviation values.

Under the conditions used, unchallenged *S. aureus* cells reached the end of the exponential phase after ca. 6.3 h (**Figure 1A**). When mid-exponential cells were challenged with 50 and 100 mg/L of vancomycin and teicoplanin, the typical biphasic killing curves with a plateau or a slow decrease in CFUs were obtained in the present study, indicating the survival of the subpopulation of persister cells (**Figure 1B**). During the fast killing phase, observed with up to 8 h of exposure, the number of survivor cells decreased 4–9 orders of magnitude in comparison with the initial number of viable cells. A plateau in the number of CFU was observed for each curve from 8 h of exposure onward. However, the biphasic killing curves were not superimposable, depending both on the antibiotic type and concentration.

To further assess the susceptibility of exponentially growing *S. aureus* cultures to vancomycin and teicoplanin and the nature of the surviving cells, cell viability was determined under the same conditions by fluorescent microscopy and image analysis. This was also used to assess if persister cells could be isolated from the dead biomass. The bacterial viability kit used in the microscopy assay distinguishes viable from non-viable cells based on the condition of the cell membrane: SYTO^®^9 stains all bacteria whilst propidium iodide only stains bacteria with damaged membranes.

**FIGURE 2:**
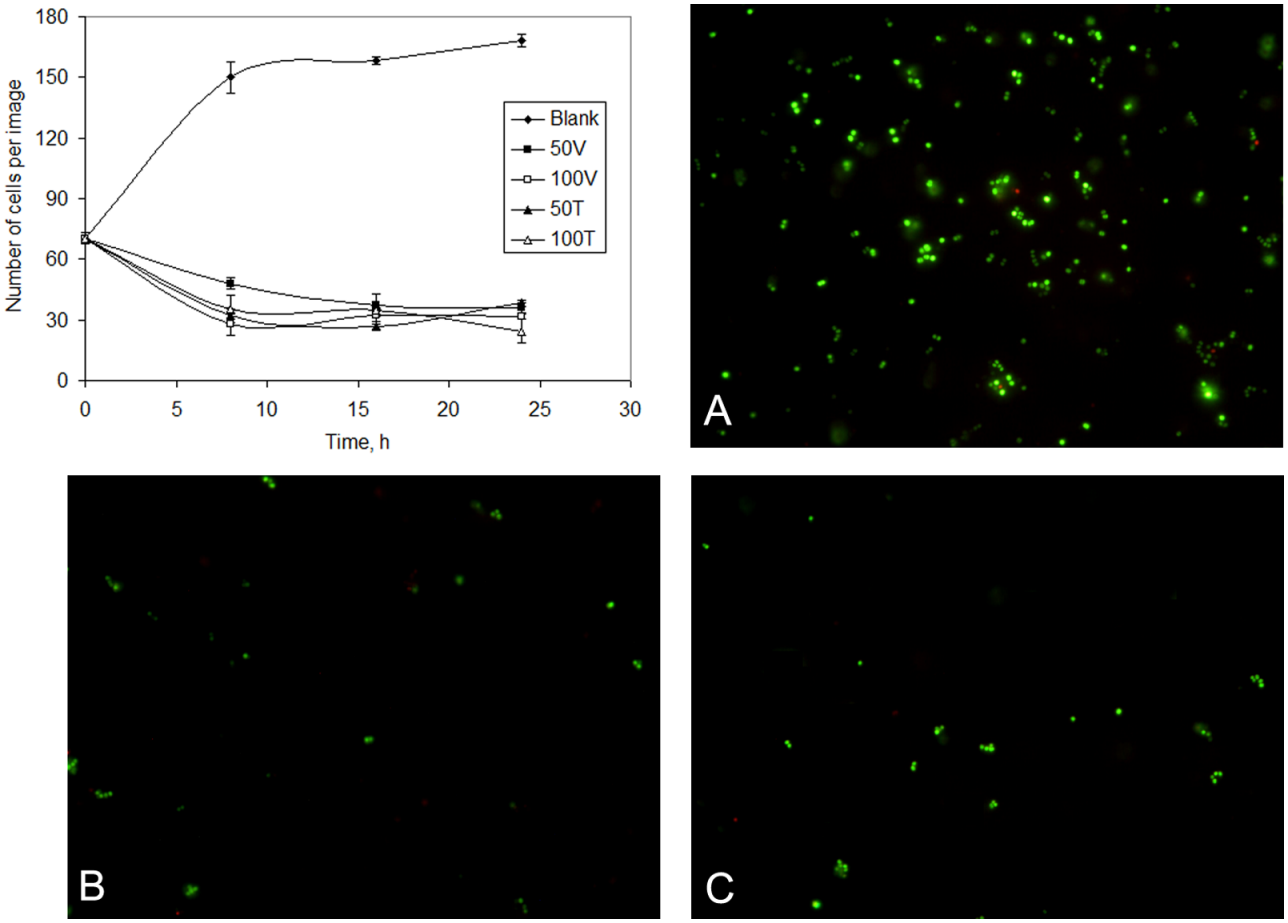
**Number of cells of *S. aureus* cells observed per image (chart) in unchallenged (blank) and following different periods of exposure to 50 and 100 mg/L teicoplanin (50T and 100T) and vancomycin (50V and 100V).** Cultures observed by fluorescence microscopy during the exponential phase **(A)**, and after exposure to 100 mg/L teicoplanin **(B)** and vancomycin **(C)** for 24 h. Viable cells are stained green whilst non-viable cells are stained red by the LIVE/DEAD viability kit.

The number of cells per image increased with time in the blank assay (from 70 ± 2 to 169 ± 12 cells/image) but it decreased to 36 ± 3 and 32 ± 5 cells/image after 24 h exposure to 50 and 100 mg/L vancomycin, respectively, and to 38 ± 6 and 24 ± 4 cells/image in the populations exposed to the same concentrations of teicoplanin (**Figure 2**). After 5 h of exposure, high cell viability values were observed in all cultures: 99.6% of the cells were viable in the blank culture whilst those challenged with antibiotics presented cell viabilities higher than 74.8% (data not shown). After 24 h, more than 86% of the cells observed per image were also viable (**Figure 2**). Combining the information of the decrease in cell number per image and high cell viability with time, this indicates that the high antibiotic concentration used should have caused cell lysis of non-persistent cells. However, according to the killing curves the number of survivor cells decreased 4–9 orders of magnitude after 8–24 h of exposure, in comparison with the initial number of cells in the culture, whilst fluorescence microscopy showed a twofold decrease. The number of viable cells was thus much higher than the number of cells that were able to form colonies. VBNC cells are similar to persister cells since they are genetically identical to susceptible cells, present a stress-tolerant phenotype and are in a non-growing state (Li et al., 2014). Exponentially growing cultures of wild-type *Escherichia coli* treated with ampicillin also presented almost 100-fold more VBNC cells than persisters (Orman and Brynildsen, 2013b). As mentioned by the authors of that study, under normal culturing conditions, VBNC cells are in larger proportion than persister cells in untreated samples, and measurement of persister physiology should be based on their ability to tolerate antibiotics and resume growth on drug-free media.

A method to isolate persisters was proposed by Keren et al. (2004) and included the addition of high concentration of antibiotics to cell cultures to cause the lysis of part of the population. The remaining persister fraction could be recovered by sedimentation through centrifugation. Antibiotics such as vancomycin cause bacterial autolysis of subpopulations of *S. aureus*, as observed by the live/dead staining method (Hsu et al., 2011). Recently, Orman and Brynildsen (2013b) disputed the conclusions of the β-lactam isolation method used by Keren et al. (2004) to isolate persisters since the method produces several orders of magnitude more VBNC than persister cells.

In the present study, the high concentrations of vancomycin and teicoplanin used (well above the MICs) also resulted in cell lysis of growing cells and in the isolation of a subpopulation of alive cells, thus allowing the biochemical analysis described below. In fact, recent studies have defended that VBNC and persister cells coexist and are induced by the same conditions, although only the latter cells resume growth in fresh medium (Li et al., 2014; Ayrapetyan et al., 2015b).

To confirm the previously reported reversible phenotypic character of persisters (Balaban et al., 2004; Rainey et al., 2011) the survivor cells (after exposure to high antibiotic concentrations) were collected and placed in fresh MHB containing no drug. Contrarily to VBNC cells, persister cells are able to resume growth when placed in fresh media (Li et al., 2014). All cultures, previously exposed to antibiotics for 3, 4, 5, or 6 h (non-dormant cells according to the killing curve), and for 8, 16, and 24 h (corresponding to the “plateau stage” of the killing curve), were able to resume growth, although at lower rates than non-exposed cells (**Figures 1C,D**). The doubling time increased from 0.6 h for non-exposed cells to 3.6 h for cells exposed to 100 mg/L vancomycin for 6 h, whilst the doubling time for cells exposed for 6 h to the same concentration of teicoplanin was 2.3 h. However, the *lag* phases were in general 33% longer after exposure to teicoplanin, when compared to that of cultures exposed to vancomycin at the same concentrations. No clear exponential phases were observed in the growth curves of cells previously exposed to each antibiotic for 8, 16 and 24 h, during the 27 h the growth was monitored (**Figures 1C,D**). The regrowth of the survivor cells in fresh MHB in the present study was also monitored by fluorescence microscopy. The increase in optical density (**Figures 1C,D**) was confirmed by an increase in the number of cells per image along time, with no aggregation of the cells being observed (data not shown).

### Antibiotic Induced Adaptations at the Cellular Envelop

#### Fatty Acid Composition

Several studies have reported that extensive modifications in the FA composition of the cytoplasmic membrane of VBNC cells are essential for entry into this state and for the maintenance of membrane potential (Porter et al., 1995; Day and Oliver, 2004; Oliver, 2005). To elucidate the physiological changes/mechanisms at the level of the cellular envelop of the cells exposed to high antibiotic concentrations, the differences in the FA profile of the phospholipids of *S. aureus* cells, before and after different periods of exposure to vancomycin and teicoplanin, were analyzed. Simultaneously, the net surface charge of the cells was also determined.

**FIGURE 3:**
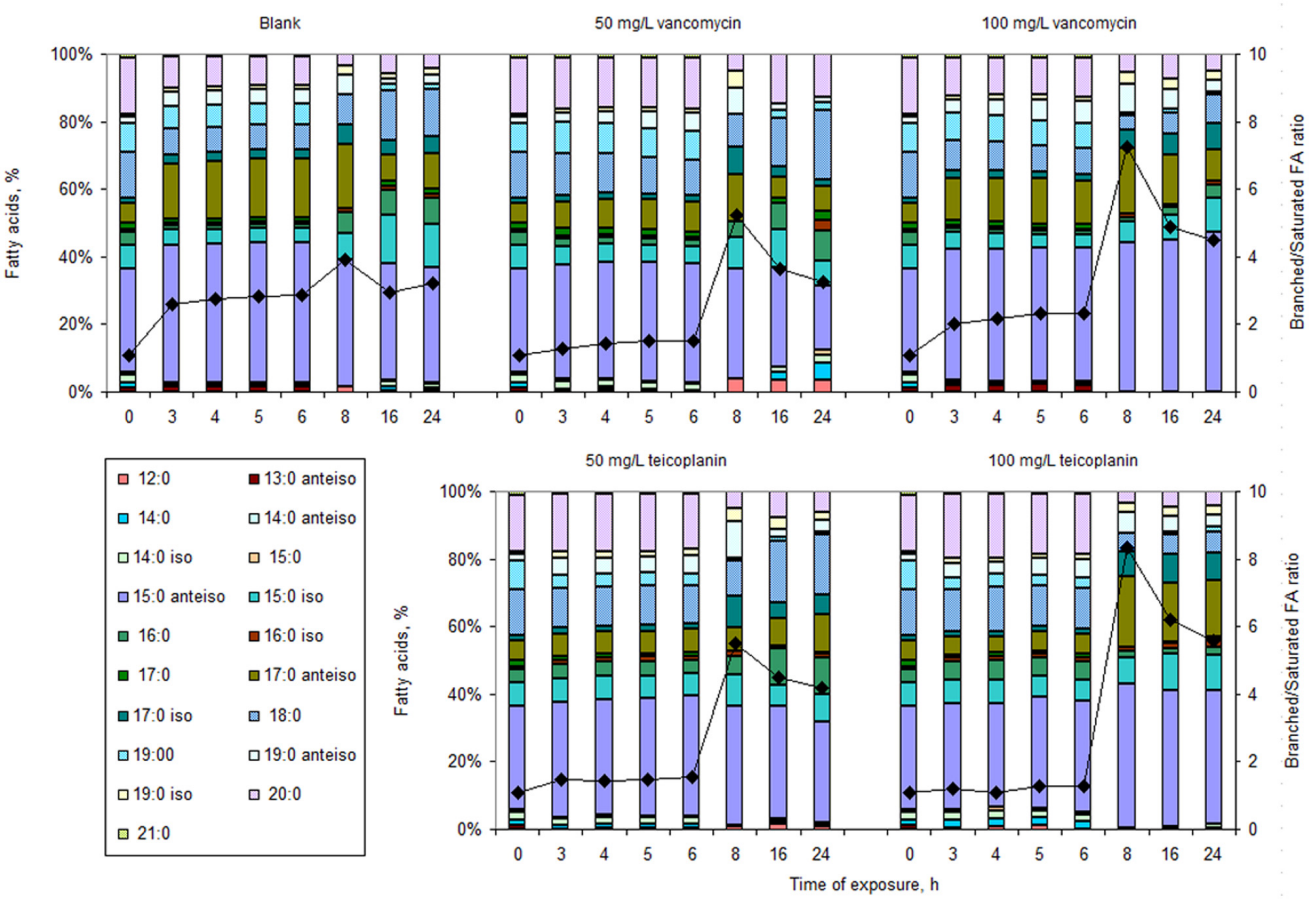
**Fatty acid (FA) composition of cells (bars) and corresponding branched/saturated FA ratio (diamonds) growing in MHB in the absence (Blank) or presence of 50 and 100 mg/L vancomycin and teicoplanin for 3, 4, 5, 6, 8, 16, and 24 h**.

Under the tested conditions, the main FAs in *S. aureus* cells were the saturated 16:0, 18:0, 19:0 and 20:0, and the branched 15:0 *iso*, 15:0 *anteiso*, 17:0 *iso*, 17:0 *anteiso* and 19:0 *anteiso*, representing more than 90% of the total lipids (**Figure 3**).

Cells in drug-free MHB, and exposed to both vancomycin and teicoplanin for up to 6 h, decreased the percentage of saturated FA with time: ca. 47% for non-stressed cells; ca. 18 and 38% and 19 and 9% for cells in the presence of 50 and 100 mg/mL vancomycin and teicoplanin, respectively (**Figure 3**). However, for each exposure time, the percentage of saturated FA was always higher in cells exposed to antibiotics than in those which were not: up to 55% higher after 6 h in the presence of 50 mg/L of both vancomycin and teicoplanin and up to 70% higher in the presence of 100 mg/L teicoplanin. Concomitantly to the observed decrease in the percentage of saturated FA, the cells increased the amount of branched FA with time: drug-free cells presented an increase of ca. 44%, whilst cells exposed up to 6 h to 50 and 100 mg/L of vancomycin and teicoplanin showed an increase of, respectively, 16 and 35, and 18 and 9%. The amount of branched chain FA was, however, lower in cells exposed to the antibiotics than in cells in drug-free medium. Besides, the *iso*/*anteiso* FA ratio decreased with exposure time: ca. 20 and 50% for cells exposed to teicoplanin and vancoymcin, respectively. Moreover, most of the alterations occurred during the first 3 h of exposure, indicating relatively fast mechanisms of response to the presence of antibiotic. Cells exposed for up to 6 h to antibiotics should thus present a less fluid membrane than cells that were not in the presence of antibiotics.

On the contrary, from 8 h of exposure onward, when the killing curve reached the “plateau phase,” the survivor cells under all conditions tested presented a decreased percentage of saturated FA and increased percentage of branched FA than cells unchallenged and those exposed for shorter periods (**Figure 3**). However, the percentages attained were dependent on the antibiotic concentration, with cells exposed to 100 mg/L of either antibiotic presenting half the percentage of saturated FA and 20% higher percentage of branched FA than unchallenged cells. Furthermore, the *iso*/*anteiso* FA ratio decreased ca. 1.1 and 1.5-fold for cells exposed to teicoplanin and vancomycin, respectively. These cells thus responded to the antibiotics by increasing the fluidity of the cellular membrane.

By comparing both **Figures 1B** and **3**, it is possible to observe that the typical plateau in the biphasic killing curve corresponding to dormant cells, attained after 8 h of exposure to both concentrations and antibiotics tested (**Figure 1B**), was associated with significant alterations in the FA profile of the exposed cells (**Figure 3**). Adjustments in the lipid composition of the cell membrane should be a phenotypic adaptation of the survivor cells.

To assess the transient nature of the survivor cells, after a period of 3, 4, 5, 6, 8, 16, and 24 h in the presence of the two concentrations of vancomycin and teicoplanin tested, the cells were collected, washed and placed in fresh drug-free MHB. According to the classical definition of persister and VBNC cells, only persister cells should grow when placed in drug-free media.

**FIGURE 4:**
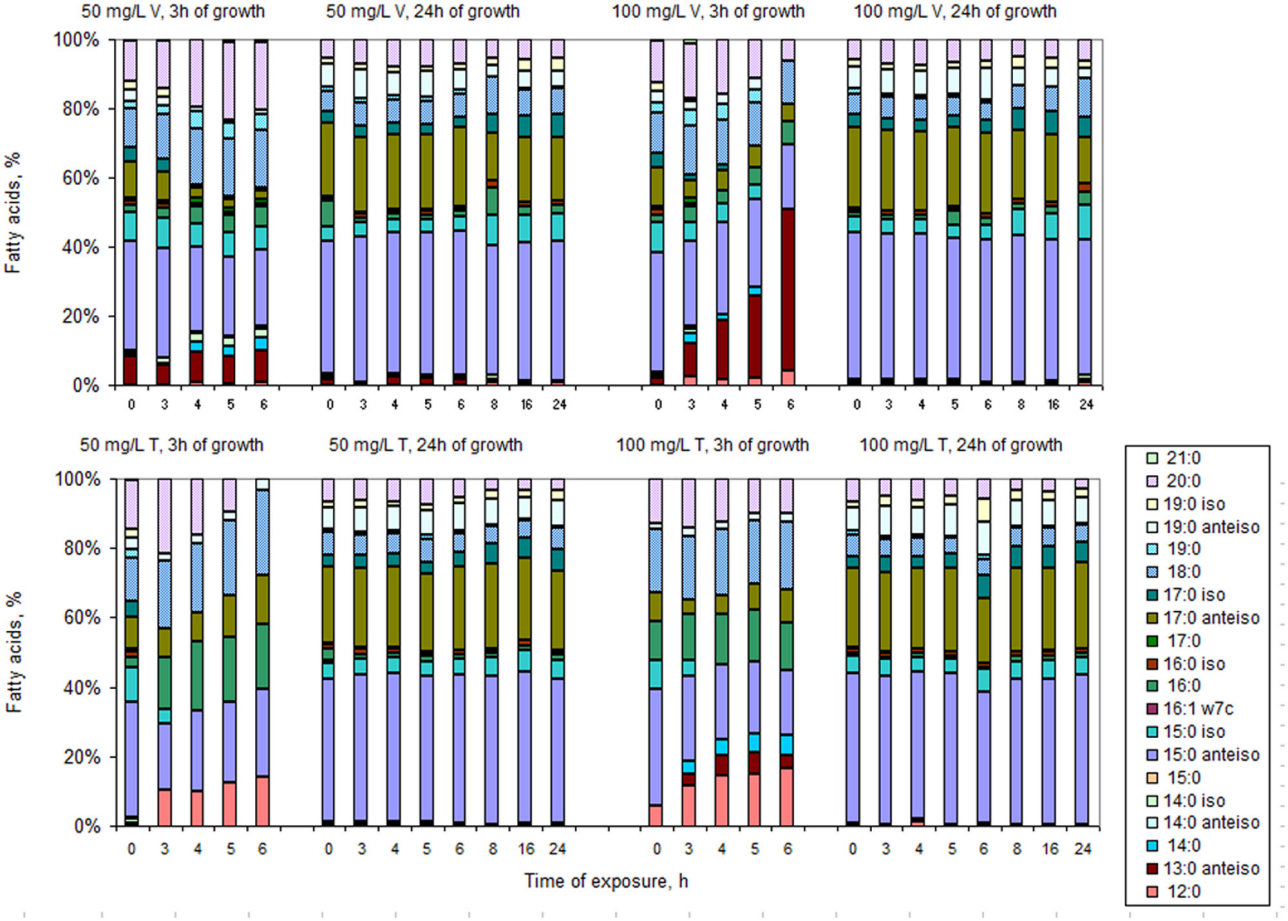
**Fatty acid composition of cells growing for 3 and 24 h in drug-free MHB after exposure to 50 and 100 mg/L vancomycin (V) and teicoplanin (T) for 3, 4, 5 and 6, 8, 16, and 24 h**.

After 3 h of growth in fresh media, the cells that had been exposed to 50 mg/L teicoplanin for 6 h or less presented different lipid composition from those exposed to 100 mg/L teicoplanin and from those exposed to the two concentrations of vancomycin (**Figure 4**). When the survivor cells (**Figure 1B**) isolated after 8, 16, and 24 h of exposure to vancomycin and teicoplanin were collected, washed, and placed in drug-free MHB, they grew at a much slower rate than those pre-exposed for 6 h or less (**Figures 1C,D**). After 3 h of growth in fresh media, the number of cells in the cultures derived from the persister cells was insufficient to allow good and reliable FAMEs analysis. Therefore, the FA composition of these cells is presented after 24 h of growth (**Figure 4**), when the cells were actively growing (**Figures 1C,D**).

**FIGURE 5:**
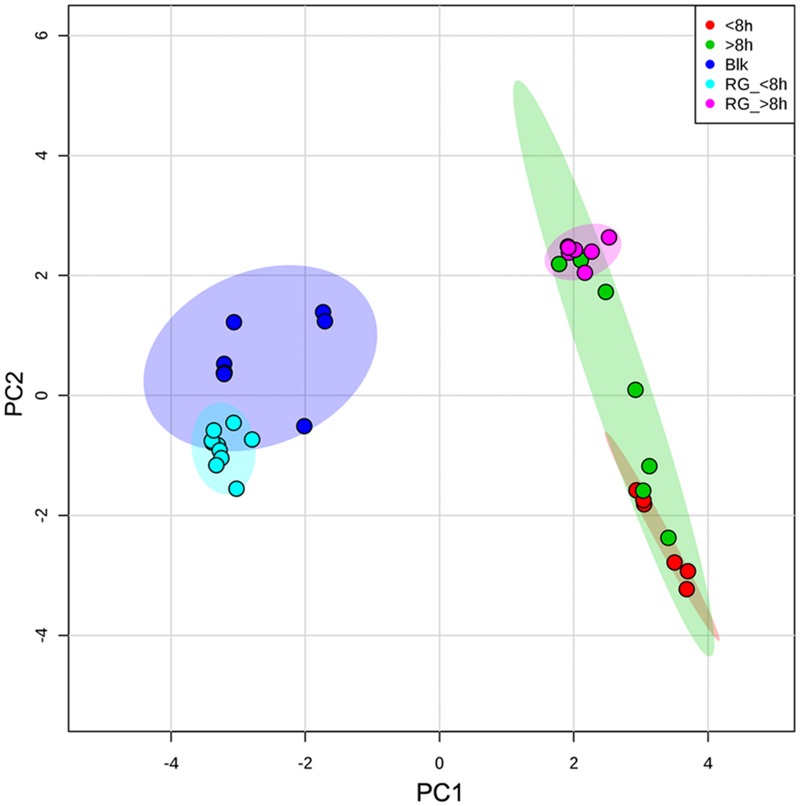
**Scores plot between PC1 and PC2.** The data are represented according to the time of exposure to antibiotics (<8 and >8 h) and the duration of exposure to antibiotics prior to growth on drug-free medium (RG_ < 8 h; RG_ > 8 h). The colored areas represent 95% confidence intervals.

Curiously, the cells presented a “memory” of the pre-exposure condition and, after 24 h, the cells presented a FA composition (**Figure 4**) similar to that of cells exposed for 8–24 h to each antibiotic (**Figure 3**), regardless of the duration of the exposure. Comparison of the FA composition of *S. aureus* derived from persister cells exposed to both 50 and 100 mg/L of vancomycin and teicoplanin, and the composition of cells exposed for less than 8 h, indicates that the major changes were observed in the percentage of *anteiso* FA and long chain FA. Both the daughters of persister cells and the survivor cells (exposed to antibiotics for 8–24 h) presented FA profiles that lead to membrane fluidity: low percentage of saturated and *anteiso*-branched FA. PCA could separate the FA data: blank and cells regrown on fresh media after exposure for less than 8 h could be separated from the remaining cells along PC1, with overlapping 95% confidence intervals (**Figure 5**). The samples could also be separated along PC2 by the duration of the period of exposure to the antibiotics, with cells exposed for longer periods scoring higher values on this PC. The PCA does indicate that cells exposed for less than 8 h present a FA composition similar to non-challenged cells when placed in drug-free medium. On the other hand, the FA of cells grown on fresh MHB after exposure for more than 8 h to antibiotics, presented a FA composition similar to cells exposed for 8–24 h. This further supports the memory effect observed.

#### Net Surface Charge

**FIGURE 6:**
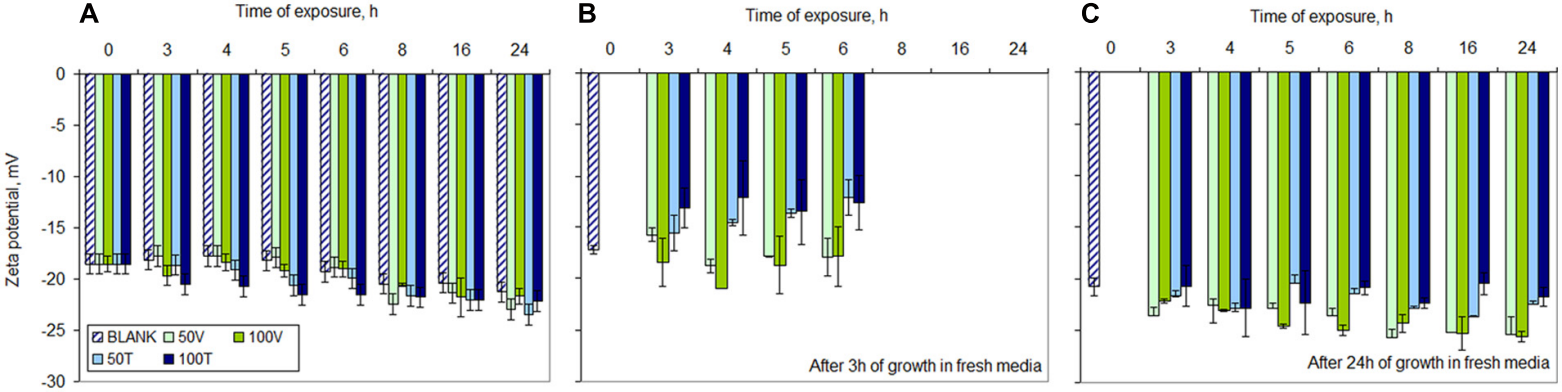
**Net surface charge of *S. aureus* cells during **(A)** exposure to 50 and 100 mg/L of vancomycin (V) and teicoplanin (T), and after 3 **(B)** and 24 h **(C)** in fresh drug-free MHB.** Blank – non-exposed cultures. Error bars represent standard deviation values.

Under no-stress conditions, *S. aureus* cells became more negatively charged with increasing age of the culture: between -18.5 ± 0.7 and -21.3 ± 1.3 mV during the 24 h of the assay (**Figure 6A**). Similarly, in the presence of vancomycin the cells presented a surface charge of ca. -21.7 ± 0.8 mV after 24 h of exposure, whilst in the presence of 50 and 100 mg/L teicoplanin the cells had a zeta potential of -23 ± 2.6 and -22.2 ± 2.1 mV, respectively. Besides, at each assayed time, cells in the presence of vancomycin and teicoplanin presented zeta potential values more negative than non-stressed cells, with the values becoming more negative with increasing antibiotic concentration (**Figure 6A**).

When the cells exposed to the two antibiotics were collected and placed in fresh drug-free MHB, the net surface charge became very similar after 3 h of growth: cells pre-exposed to 50 and 100 mg/L vancomycin presented an average of -17.6 ± 0.8 and -19.0 ± 2.0 mV, respectively, whilst cells pre-exposed to the same concentrations of teicoplanin presented values of -14.0 ± 1.1 and -12.9 ± 2.9 mV, regardless of the period of exposure (**Figure 6B**). The cultures inoculated with cells exposed to the antibiotics for 8, 16, and 24 h did not present enough cells to obtain sound and reliable zeta potential measurements after 3 h of growth in fresh MHB.

After 24 h of growth in fresh MHB, the cells pre-exposed to the antibiotics presented zeta potential values more negative than non-exposed cells: those exposed to 50 mg/L of vancomycin for 24 h presented a zeta potential value 22.3% more negative than non-exposed cells while cells exposed to 100 mg/L increased in 23.0% the negative character of their surface (**Figure 6C**). On the other hand, cells exposed to teicoplanin presented values closer to non-stressed cells: between 7.8 and 4.7% for cells exposed to 50 and 100 mg/L, respectively. Besides, while cells pre-exposed to antibiotics up to 6 h presented a time-dependent decrease of the zeta potential values, those that were exposed for 8, 16, and 24 h (corresponding to the “plateau phase” in **Figure 1B**) presented similar net surface charge values: average of -25.4 ± 0.8 and -25.0 ± 0.9 mV when the cells were exposed to 50 and 100 mg/L vancomycin; average of -22.9 ± 0.2 and -21.5 ± 0.8 mV for cells exposed to 50 and 100 mg/L teicoplanin, respectively.

### Metabolic Activity of Antibiotic Exposed Cells

To assess the metabolic state of the cells, oxygen consumption and the activity of a specific set of enzymes present in the API^®^ ZYM kit were evaluated during exposure to both vancomycin and teicoplanin.

**FIGURE 7:**
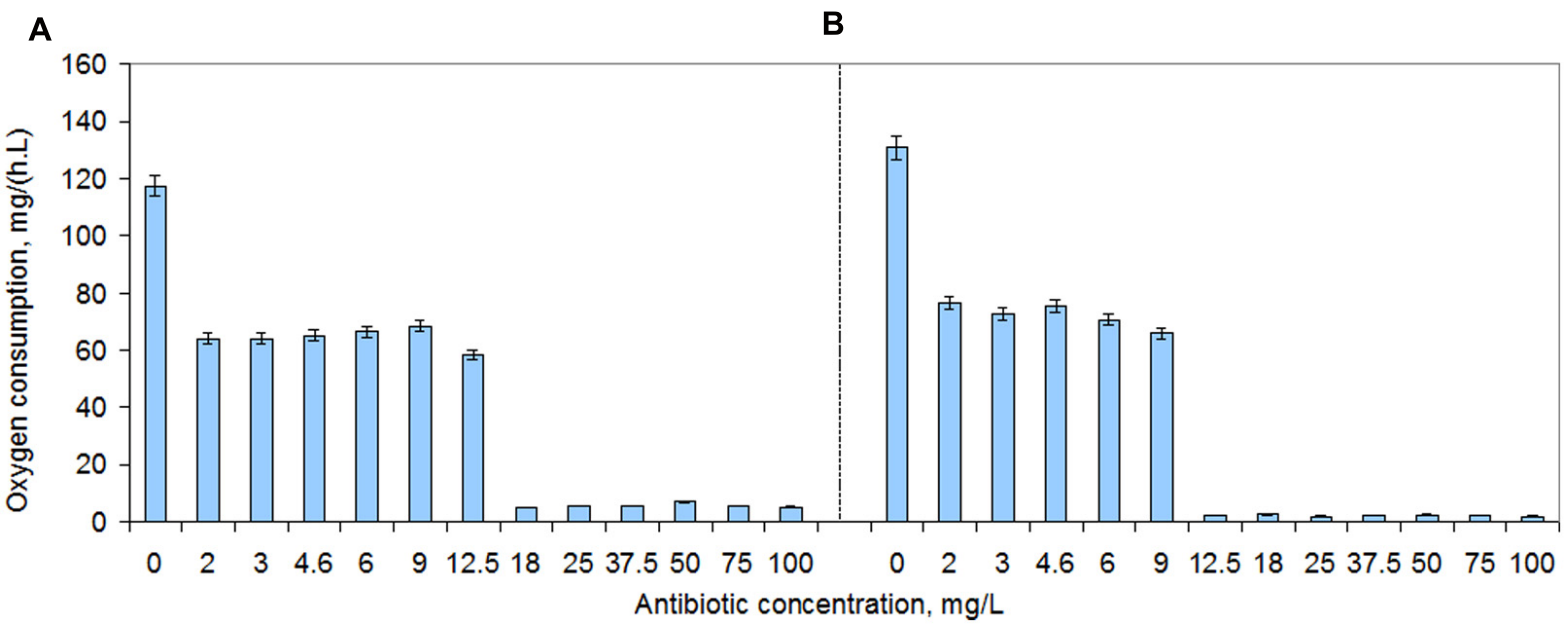
**Oxygen consumption rates of *S. aureus* cells during 2–100 mg/L vancomycin **(A)** and teicoplanin **(B)** exposure.** The plates were incubated at 37°C and 200 rpm. Error bars represent standard deviation values.

The oxygen consumption rate decreased 10-fold when the vancomycin concentration increased from 12.5 to 18 mg/L (**Figure 7A**). Similarly, the culture decreased 30-fold the oxygen consumption rate when the teicoplanin concentration increased from 9 to 12.5 mg/L (**Figure 7B**). However, the results clearly show that the cells exposed to 50 and 100 mg/L of both antibiotics were actually consuming oxygen. Since they were also able to make the necessary changes in FA composition of the cellular membranes, this is an indication that the survivor cells were metabolically active. This is in accordance to the reported metabolic active state of VBNC cells (Oliver, 2005).

**FIGURE 8:**
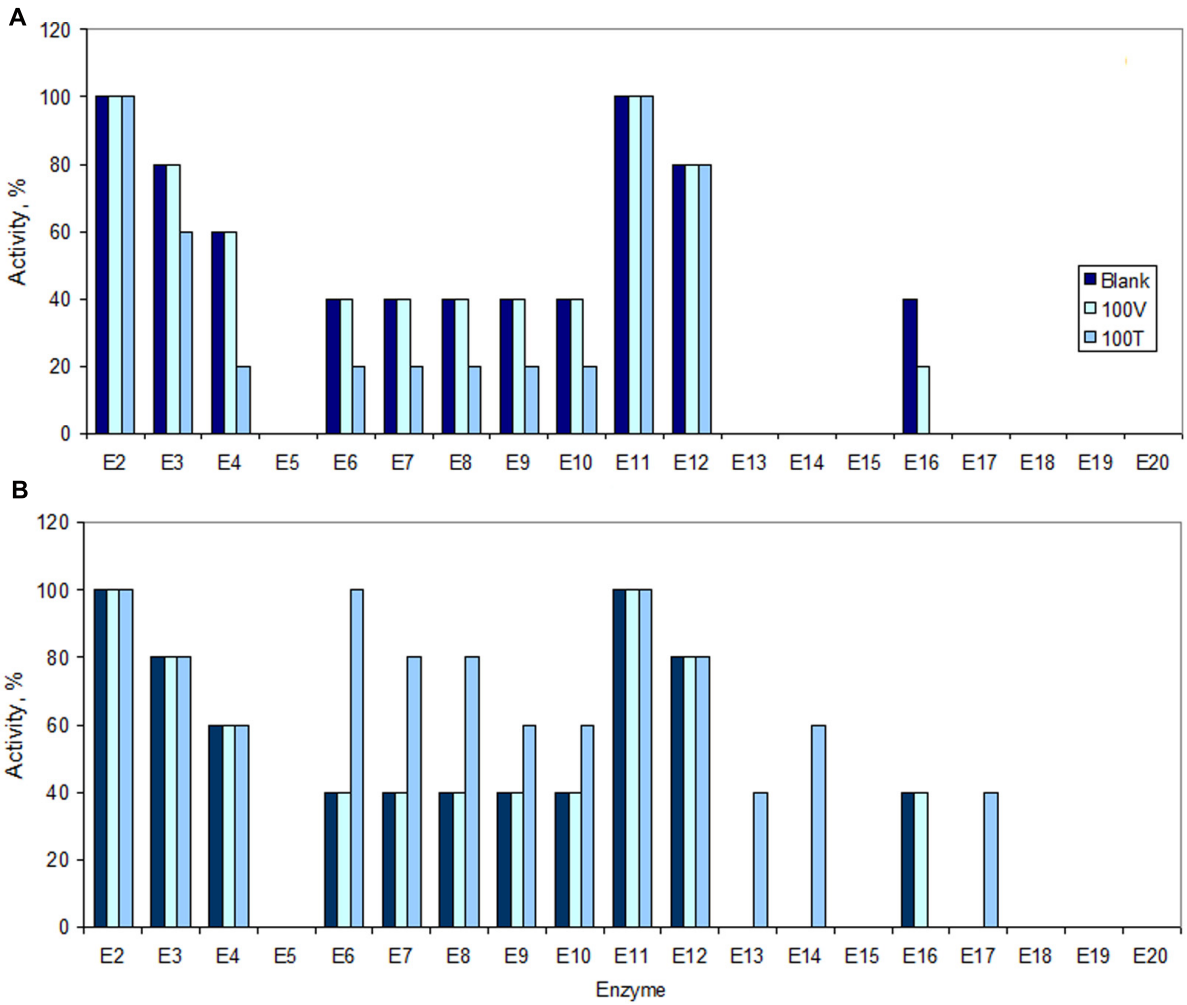
**Activity of enzymes in *S. aureus* cells under no-stress conditions (Blank), following 8 h of exposure **(A)** to 100 mg/L of vancomycin (100 V) and teicoplanin (100 T), and following 5 h of post-antibiotic growth in fresh, drug-free MHB **(B)**.** The maximum activity (100%) is referred to the maximum color intensity (5) suggested by the kit. The horizontal axis corresponds to the following enzymes: E2, alkaline phosphatase; E3, esterase (C4); E4, esterase lipase (C8); E5, lipase (C14); E6, leucine arylamidase; E7, valine arylamidase; E8, cystine arylamidase; E9, trypsin; E10, α-chymotrypsin; E11, acid phosphatase; E12, Naphtol-AS-BI-phosphohydrolase; E13, α-galactosidase; E14, β-galactosidase: E15, β-glucuronidase; E16, α-glucosidase; E17, β-glucosidase; E18, *N*-acetyl-β-glucosaminidase; E19, α-mannosidase; E20, α-fucosidase.

To further assess the metabolic activity of the cells that survived in the presence of such high concentrations of both vancomycin and teicoplanin, the activities of the 19 enzymes present in the API^®^ ZYM kit from BioMérieux were analyzed. *S. aureus* cells exposed to 100 mg/L vancomycin maintained the same level of activity of non-exposed cells with the exception for the enzyme α-glucosidase (E16) where the activity was half of that of non-stressed cells (**Figure 8A**). After exposure to 100 mg/L teicoplanin, the activity of esterase (C4) (E3) decreased to 60% whilst the activity of esterase lipase (C8) (E4), leucine (E6), valine (E7) and cystine (E8) arylamidase, trypsin (E9) and α-chymotrypsin (E10) decreased to 20%, i.e., to at least half of the activity observed in non-stressed cells. Cells exposed to teicoplanin presented also no activity of α-glucosidase (E16). Curiously, when the cells were placed in fresh drug-free MHB, the cells previously exposed to 100 mg/L vancomycin presented the same enzymatic profile as non-exposed cells after 5 h of growth, whilst those exposed to 100 mg/L teicoplanin presented increased activity in several enzymes (**Figure 8B**). The activity of E4, E9 and E10 tripled when compared to what the cells had shown during exposure to teicoplanin, and increased 4- and 5-fold in E7–E8 and E6, respectively. Besides, these cells also presented activity in α- and β-galactosidase (E13 and E14) and β-glucosidase (E17).

The enzymatic kit used contains esterases (E2–E5 and E11), peptidases and proteases (E6–E10), glycosidases (E13–E20) and a phosphohydrolase (E12). During exposure to teicoplanin, the cells thus decreased the activity of certain esterases and peptidases/proteases but a significant increase in the activity of these enzymes was observed during regrowth in fresh MHB, as well as in the activity of certain glycosidases (**Figures 8A,B**).

## Discussion

Modifications of the FA composition of the phospholipids of cellular membranes are known to be an adaptive phenotypic mechanism allowing bacterial cells to survive environmental conditions and exposure to toxic compounds (White and Frerman, 1968; Heipieper and Debont, 1994; de Carvalho et al., 2009; de Carvalho, 2012). However, almost nothing is known regarding these phenotypic modifications in persister cells. The aim of this study was to investigate if persister *S. aureus* cells presented FA compositions different from normally growing cells and what type of modifications were induced.

To assess the FA composition of persister cells, this phenotype had to be induced while the remaining cells had to be eliminated. To induce persister phenotype in *S. aureus* populations, exponentially growing cells were exposed to concentrations of both vancomycin and teicoplanin much higher than the MIC. The induction of cell lysis and the recovery of persister cells had been previously reported (Keren et al., 2004; Hsu et al., 2011; Cañas-Duarte et al., 2014). The biphasic killing kinetics, observed upon exposure to the two tested antibiotic concentrations, suggested that the majority of the susceptible, regular cells were killed whilst a small fraction of tolerant, persister cells in the antibiotic exposed populations survived from 8 h of exposure onward (**Figure 1B**). However, by comparing CFU and fluorescence microscopy data, it was possible to observe that the high concentrations of vancomycin and teicoplanin used were sufficient to cause cell lysis of normally growing cells, but the number of viable cells observed under the microscope was higher than expected according to the CFU counts.

Another well-defined dormancy state, that has been described in non-sporulating bacteria, concerns VBNC cells. These cells, first described by Xu et al. (1982) in *E. coli* and *Vibrio cholerae* cultures, are normally cultivable cells that have lost the ability to grow but remain viable. Contrarily to normal cells, which are culturable on suitable media and have the ability to form colonies on agar media, VBNC cells cannot grow or form colonies. Since the first report, this dormant state has been described in up to 85 species of bacteria and has been found to share similarities with persister cells: they are genetically identical to the remaining population, show a stress-tolerant phenotype and are in a non-growing state (Li et al., 2014). Besides, VBNC cells can exist stochastically in unstressed growing cultures and cause antibiotic failure and recurrent infections (Ayrapetyan et al., 2015a). In fact, Oliver and co-workers have defended in a recent review that persister and VBNC cells are not distinct but are part of a shared “dormancy continuum” (Ayrapetyan et al., 2015b). Other studies have also suggested that these two types of cells should be probably considered the same (Li et al., 2014; Ayrapetyan et al., 2015a).

The fact that a culture of *E. coli* in logarithmic phase contains 100-fold more VBNC cell than persisters (Orman and Brynildsen, 2013b), suggests that both VBNC and persister cells are part of a bet-hedging strategy aimed at protecting the population from lethal conditions and that persistence could be a transitory state leading to the VBNC state (Ayrapetyan et al., 2015b).

In the present study, the distinct survival rates obtained for different antibiotics/concentrations further indicates physiological heterogeneity within the survival population. Although vancomycin and teicoplanin possess a similar mechanism, since both compounds inhibit peptidoglycan synthesis by interacting with the D-alanyl-D-alanine terminal of the pentapeptide side chains of peptidoglycan precursors (Nieto and Perkins, 1971; Parenti, 1986), the death rates depended on the antibiotic and concentration used (**Figure 1B**). The heterogeneity of the survivor persister population has also been observed by other authors (Dhar and McKinney, 2007; Gefen and Balaban, 2009; Allison et al., 2011a; Lechner et al., 2012), and should be an important part of the bet-hedging strategy.

After isolation of the survivor cells, modifications observed in cellular membrane composition and in cell surface properties could thus be analyzed. The major changes occurring in bacteria are usually related to the maintenance of an appropriate membrane fluidity (Joyce et al., 1970; de Carvalho et al., 2009; de Carvalho, 2012; Mirani and Jamil, 2013). The FA present as acyl chains of phospholipids and glycolipids determine the fluidity of cellular membranes and changes in their composition allow bacterial cells to survive under a wide array of environments (Sinensky, 1974; Zhang and Rock, 2008; de Carvalho, 2012). In *S. aureus*, branched FA represent ca. 55–65% of the total FA (the major FA being 15:0 *anteiso*) and are the major determinants of membrane fluidity (Singh et al., 2008). In the present study, in the absence of antibiotics, cells increased the percentage of branched FA while decreasing the percentage of saturated FA with the age of the culture (**Figure 3**). On the other hand, during up to 6 h of antibiotic exposure, *S. aureus* promoted a decrease in the branched/saturated FA ratio when compared with non-exposed cells. Cells exposed to both antibiotics thus presented higher percentage of saturated FA and lower amount of branched chain FA than cells in drug-free MHB. The exposed cells thereby responded to the presence of antibiotics at the lipid level as bacterial cells usually respond to a number of toxic organic compounds: by decreasing the fluidity of the cellular membrane. Nevertheless, cells exposed to 100 mg/L vancomycin presented only 15–18% more saturated FA than non-stressed cells. Vancomycin and teicoplanin are structurally related but in vancomycin the two chlorinated β-hydroxytyrosine moieties are asparagine and *N*-methyl-leucine while in teicoplanin these are substituted by linked hydroxyphenyl-glycine moieties (Reynolds, 1989; Johnson et al., 1990). Besides, in teicoplanin the acyl substitute of the *N*-acylglucosamine is a FA containing 10–11 carbon atoms, resulting in a higher hydrophobicity of teicoplanin in comparison to vancomycin (Parenti, 1986). This FA acyl group is thought to anchor the antibiotic to the bacterial membrane, resulting in an increased concentration of the antibiotic at the site of the peptidoglycan biosynthesis (Beauregard et al., 1995; Westwell et al., 1995). This could be the reason why the cells also presented more changes in the presence of teicoplanin than in the presence of vancomycin.

When the survivor cells were analyzed after exposure for 8–24 h, it was found that these cells presented significant differences in the FA profile (**Figure 3**). Major changes involved a significant (1.2- to 2.1-fold) increase of the branched/saturated FA ratio. Besides, the *iso*/*anteiso* FA ratio decreased with time in the survivor cells and it was 0.5–0.9 times lower than that of non-exposed cells. Differences in the proportion of *iso* and *anteiso* FA have been observed in methicillin sensitive and resistant *S. aureus* strains (Pinchuk et al., 1983) and a significant increase in the *anteiso*/*iso* FA ratio accompanied by a decrease in saturated FA was observed in a daptomycin resistant strain when compared to the sensitive strain (Mishra et al., 2014). When the enzyme complex branched-chain α-keto acid dehydrogenase, which catalyzes the early stages of branched-chain FA in *S. aureus*, was inactivated, the resulting mutant presented reduced proportions of branched chain FA, was more susceptible to stress conditions and showed reduced adherence to eukaryotic cells (Singh et al., 2008). The survivor cells in the present study thus responded to the high concentrations of antibiotics similarly: by decreasing the order of the cellular membrane (by decreasing both the saturated and *iso* branched FA) and increasing the membrane fluidity. Day and Oliver (2004) also found that changes in the FA composition, aimed at the maintenance of membrane fluidity in *Vibrio vulnificus* cells entering the VBNC state, was a factor for this physiological response. Besides, cells with inhibited FA synthesis did not survive, thus supporting the importance of an active FA metabolism in cells entering the VBNC state.

The most curious result of this study was, however, the evidence that survivor cells presented a “memory” of the conditions to which they were exposed after growth in fresh, drug-free MHB (**Figures 4** and **5**). According to the classical definition of persister and VBNC cells, only persister cells can resume growth in fresh medium and so in the present study, regrowth should result from the persister fraction of the survivor cells. Nevertheless, favorable growth conditions with proper carbon and energy sources, in an ideal stoichiometric ratio to inorganic elements, are known to allow the resuscitation of VBNC cells (Ramamurthy et al., 2014).

After 3 h of growth in drug-free medium, the pre-exposed cells still presented a FA composition that was different from that of non-exposed cells, whilst, even after 24 h, the daughters of persister cells still presented a FA composition similar to the composition of the cells that survived 8–24 h exposure to high concentrations of antibiotics. It has been shown that some persisters have a reduced growth rate that is passed to the following generations over several divisions before becoming “normal” cells, which constitutes a non-genetic inheritance (Gefen and Balaban, 2009). This was also observed in this study when the persister cells were placed in drug-free medium (**Figures 1C,D**). Non-genetically transmissible phenotypes can be induced by environmental conditions and provide “transgenerational plasticity” (Bonduriansky et al., 2012) allowing the reduction of *lag* phases following the first exposure because the offspring will express the best phenotype (Jablonka et al., 1995), but could be disadvantageous if environmental fluctuations are fast (Paenke et al., 2007). In the present case, the cells made an “investment” in transmitting the phenotype allowing their offspring to survive in very high concentrations of vancomycin and teicoplanin, resulting in longer *lag* phases when the cells where placed in fresh MHB without the stress agents.

*Staphylococcus aureus* cells also presented a more negative net surface charge with both time of exposure and antibiotic concentration (**Figure 6**). This could contribute to a repulsion mechanism toward the negatively charged (at neutral pH) teicoplanin molecule but should have the opposite effect toward the positively charged vancomycin which would that way be attracted to a more negative membrane. In fact, it has been shown that heterogeneous Vancomycin-Intermediate-*S. aureus* (hVISA) and Vancomycin-Intermediate-*S. aureus* (VISA) strains present in common an increased positive cell wall charge responsible for the repulsion of vancomycin (Cafiso et al., 2012), as well as *S. aureus* strains resistant to daptomycin and vancomycin (Cui et al., 2010). However, in this study, even the offspring of cells exposed to vancomycin and grown on fresh drug-free MHB presented net surfaces charges 20% more negative than non-challenged cells (**Figure 6**). This indicates that phenotypic changes related to persistence should use mechanisms different from those used by resistant strains.

Both persister and VBNC cells are usually considered as being in a dormant state. However, the *S. aureus* survivor cells tested consumed oxygen during exposure to both vancomycin and teicoplanin (**Figure 7**) and presented enzymatic activity of enzymes related to lipid, protein and saccharide metabolism (**Figure 8**). Other studies have recently suggested that although dormant, persister cells are primed for metabolite uptake, central metabolism and respiration (Allison et al., 2011b; Orman and Brynildsen, 2013b; Prax and Bertram, 2014). The metabolic active state of VBNC cells has also been reported (Oliver, 2005).

In the present study, (i) the number of VBNC was much larger than the number of persister cells, (ii) the FA composition of the cells after regrowth in fresh-drug free media is similar to the FA composition of the parent cells, and (iii) only persister cells should resume growth upon placed in new media. This indicates that VBNC and persister share the same phenotypic response to antibiotics at the lipid level.

## Conflict of Interest Statement

The authors declare that the research was conducted in the absence of any commercial or financial relationships that could be construed as a potential conflict of interest.

## References

[B1] Allison K. R. Brynildsen M. P. Collins J. J. (2011a). Heterogeneous bacterial persisters and engineering approaches to eliminate them. *Curr. Opin. Microbiol.* 14 593–598. 10.1016/j.mib.2011.09.002 21937262 PMC3196368

[B2] Allison K. R. Brynildsen M. P. Collins J. J. (2011b). Metabolite-enabled eradication of bacterial persisters by aminoglycosides. *Nature* 473 216–220. 10.1038/nature10069 21562562 PMC3145328

[B3] Ayrapetyan M. Williams T. C. Baxter R. Oliver J. D. (2015a). Viable but nonculturable and persister cells coexist stochastically and are induced by human serum. *Infect. Immun.* 83 4194–4203. 10.1128/IAI.00404-15 26283335 PMC4598401

[B4] Ayrapetyan M. Williams T. C. Oliver J. D. (2015b). Bridging the gap between viable but non-culturable and antibiotic persistent bacteria. *Trends Microbiol.* 23 7–13. 10.1016/j.tim.2014.09.004 25449050

[B5] Balaban N. Q. Merrin J. Chait R. Kowalik L. Leibler S. (2004). Bacterial persistence as a phenotypic switch. *Science* 305 1622–1625. 10.1126/science.1099390 15308767

[B6] Bascomb S. Manafi M. (1998). Use of enzyme tests in characterization and identification of aerobic and facultatively anaerobic Gram-positive cocci. *Clin. Microbiol. Rev.* 11 318–340. 9564566 10.1128/cmr.11.2.318 PMC106835

[B7] Beauregard D. A. Williams D. H. Gwynn M. N. Knowles D. J. (1995). Dimerization and membrane anchors in extracellular targeting of vancomycin group antibiotics. *Antimicrob. Agents Chemother.* 39 781–785. 10.1128/AAC.39.3.781 7793894 PMC162627

[B8] Bigger J. W. (1944). Treatment of staphylococcal infections with penicillin by intermittent sterilisation. *Lancet* 2 497–500. 10.1016/S0140-6736(00)74210-3

[B9] Bonduriansky R. Crean A. J. Day T. (2012). The implications of nongenetic inheritance for evolution in changing environments. *Evol. Appl.* 5 192–201. 10.1111/j.1752-4571.2011.00213.x 25568041 PMC3353344

[B10] Cafiso V. Bertuccio T. Spina D. Purrello S. Campanile F. Di Pietro C. (2012). Modulating activity of vancomycin and daptomycin on the expression of autolysis cell-wall turnover and membrane charge genes in hVISA and VISA strains. *PLoS ONE* 7:e29573 10.1371/journal.pone.0029573 PMC3253798 22253738

[B11] Cañas-Duarte S. J. Restrepo S. Pedraza J. M. (2014). Novel protocol for persister cells isolation. *PLoS ONE* 9:e88660 10.1371/journal.pone.0088660 PMC3931647 24586365

[B12] CLSI (2014). *Performance Standards for Antimicrobial Susceptibility Testing; Twenty-Fourth Informational Supplement. CLSI Document M100-S24.* Wayne, PA: Clinical and Laboratory Standards Institute.

[B13] Costerton J. W. Stewart P. S. Greenberg E. P. (1999). Bacterial biofilms: a common cause of persistent infections. *Science* 284 1318–1322. 10.1126/science.284.5418.1318 10334980

[B14] Cui L. Isii T. Fukuda M. Ochiai T. Neoh H.-M. Camargo I. L. B. D. C. (2010). An RpoB mutation confers dual heteroresistance to daptomycin and vancomycin in *Staphylococcus aureus*. *Antimicrob. Agents Chemother.* 54 5222–5233. 10.1128/AAC.00437-10 20837752 PMC2981288

[B15] Darley E. S. R. MacGowan A. P. (2004). The use and therapeutic drug monitoring of teicoplanin in the UK. *Clin. Microbiol. Infect.* 10 62–69. 10.1111/j.1469-0691.2004.00747.x 14706088

[B16] Dawson C. C. Intapa C. Jabra-Rizk M. A. (2011). “Persisters”: survival at the cellular level. *PLoS Pathog.* 7:e1002121 10.1371/journal.ppat.1002121 PMC3145784 21829345

[B17] Day A. P. Oliver J. D. (2004). Changes in membrane fatty acid composition during entry of *Vibrio vulnificus* into the viable but nonculturable state. *J. Microbiol.* 42 69–73. 15357297

[B18] de Carvalho C. C. C. R. (2012). Adaptation of *Rhodococcus erythropolis* cells for growth and bioremediation under extreme conditions. *Res. Microbiol.* 163 125–136. 10.1016/j.resmic.2011.11.003 22146587

[B19] de Carvalho C. C. C. R. Pons M. N. da Fonseca M. M. R. (2003). Principal components analysis as a tool to summarise biotransformation data: influence on cells of solvent type and phase ratio. *Biocatal. Biotrans.* 21 305–314. 10.1080/10242420310001630146

[B20] de Carvalho C. C. C. R. Wick L. Heipieper H. J. (2009). Cell wall adaptations of planktonic and biofilm *Rhodococcus erythropolis* cells to growth on C5 to C16 n-alkane hydrocarbons. *Appl. Microbiol. Biotechnol.* 82 311–320. 10.1007/s00253-008-1809-3 19096838

[B21] de Groote V. N. Verstraeten N. Fauvart M. Kint C. I. Verbeeck A. M. Beullens S. (2009). Novel persistence genes in *Pseudomonas aeruginosa* identified by high-throughput screening. *FEMS Microbiol. Lett.* 297 73–79. 10.1111/j.1574-6968.2009.01657.x 19508279

[B22] Dhar N. McKinney J. D. (2007). Microbial phenotypic heterogeneity and antibiotic tolerance. *Curr. Opin. Microbiol.* 10 30–38. 10.1016/j.mib.2006.12.007 17215163

[B23] Gefen O. Balaban N. Q. (2009). The importance of being persistent: heterogeneity of bacterial populations under antibiotic stress. *FEMS Microbiol. Rev.* 33 704–717. 10.1111/j.1574-6976.2008.00156.x 19207742

[B24] Goss C. H. Muhlebach M. S. (2011). Review: *Staphylococcus aureus* and MRSA in cystic fibrosis. *J. Cyst. Fibros* 10 298–306. 10.1016/j.jcf.2011.06.002 21719362

[B25] Grant S. S. Hung D. T. (2013). Persistent bacterial infections, antibiotic tolerance, and the oxidative stress response. *Virulence* 4 273–283. 10.4161/viru.23987 23563389 PMC3710330

[B26] Gupta A. Biyani M. Khaira A. (2011). Vancomycin nephrotoxicity: myths and facts. *Neth. J. Med.* 69 379–383. 21978980

[B27] Hansen S. Lewis K. Vulic M. (2008). Role of global regulators and nucleotide metabolism in antibiotic tolerance in *Escherichia coli*. *Antimicrob. Agents Chemother.* 52 2718–2726. 10.1128/AAC.00144-08 18519731 PMC2493092

[B28] Heipieper H. J. Debont J. A. M. (1994). Adaptation of *Pseudomonas* putida S12 to ethanol and toluene at the level of fatty acid composition of membranes. *Appl. Environ. Microbiol.* 60 4440–4444. 7811084 10.1128/aem.60.12.4440-4444.1994 PMC202003

[B29] Hofsteenge N. Van Nimwegen E. Silander O. K. (2013). Quantitative analysis of persister fractions suggests different mechanisms of formation among environmental isolates of *E. coli*. *BMC Microbiol.* 13:25 10.1186/1471-2180-13-25 PMC3682893 23379956

[B30] Hsu C.-Y. Lin M.-H. Chen C.-C. Chien S.-C. Cheng Y.-H. Su I. N. (2011). Vancomycin promotes the bacterial autolysis, release of extracellular DNA, and biofilm formation in vancomycin-non-susceptible *Staphylococcus aureus*. *FEMS Immunol. Med. Microbiol.* 63 236–247. 10.1111/j.1574-695X.2011.00846.x 22077227

[B31] Jablonka E. Oborny B. Molnar I. Kisdi E. Hofbauer J. Czaran T. (1995). The adaptive advantage of phenotypic memory in changing environments. *Philos. T. Roy. Soc. B.* 350 133–141. 10.2307/56329 8577857

[B32] Johnson A. P. Uttley A. H. Woodford N. George R. C. (1990). Resistance to vancomycin and teicoplanin: an emerging clinical problem. *Clin. Microbiol. Rev.* 3 280–291. 2143434 10.1128/cmr.3.3.280 PMC358160

[B33] Johnson P. J. T. Levin B. R. (2013). Pharmacodynamics, population dynamics, and the evolution of persistence in *Staphylococcus aureus*. *PLoS Genet.* 9:e1003123 10.1371/journal.pgen.1003123 PMC3536638 23300474

[B34] Jones T. Yeaman M. R. Sakoulas G. Yang S.-J. Proctor R. A. Sahl H.-G. (2008). Failures in clinical treatment of *Staphylococcus aureus* infection with daptomycin are associated with alterations in surface charge, membrane phospholipid asymmetry, and drug binding. *Antimicrob. Agents Chemother.* 52 269–278. 10.1128/AAC.00719-07 17954690 PMC2223911

[B35] Joyce G. H. Hammond R. K. White D. C. (1970). Changes in membrane lipid composition in exponentially growing *Staphylococcus aureus* during the shift from 37 to 25 C. *J. Bacteriol.* 104 323–330. 5473899 10.1128/jb.104.1.323-330.1970 PMC248217

[B36] Keren I. Shah D. Spoering A. Kaldalu N. Lewis K. (2004). Specialized persister cells and the mechanism of multidrug tolerance in *Escherichia coli*. *J. Bacteriol.* 186 8172–8180. 10.1128/jb.18624.8172-8180.2004 15576765 PMC532439

[B37] Lechner S. Lewis K. Bertram R. (2012). *Staphylococcus aureus* persisters tolerant to bactericidal antibiotics. *J. Mol. Microbiol. Biotechnol.* 22 235–244. 10.1159/000342449 22986269 PMC3518770

[B38] Lewis K. (2001). Riddle of biofilm resistance. *Antimicrob. Agents Chemother.* 45 999–1007. 10.1128/aac.45.4.999-1007.2001 11257008 PMC90417

[B39] Lewis K. (2007). Persister cells, dormancy and infectious disease. *Nat. Rev. Microbiol.* 5 48–56. 10.1038/nrmicro1557 17143318

[B40] Lewis K. (2010). Persister cells. *Annu. Rev. Microbiol.* 64 357–372. 10.1146/annurev.micro.112408.134306 20528688

[B41] Li L. Mendis N. Trigui H. Oliver J. D. Faucher S. P. (2014). The importance of the viable but non-culturable state in human bacterial pathogens. *Front. Microbiol.* 5:258 10.3389/fmicb.2014.00258 PMC4040921 24917854

[B42] Marques M. P. C. Walshe K. Doyle S. Fernandes P. de Carvalho C. C. C. R. (2012). Anchoring high-throughput screening methods to scale-up bioproduction of siderophores. *Process Biochem.* 47 416–421. 10.1016/j.procbio.2011.11.020

[B43] Mirani Z. A. Jamil N. (2013). Effect of vancomycin on the cytoplasmic membrane fatty acid profile of vancomycin-resistant and -susceptible isolates of *Staphylococcus aureus*. *J. Infect. Chemother.* 19 24–33. 10.1007/s10156-012-0447-y 22821354

[B44] Mishra N. N. Bayer A. S. Weidenmaier C. Grau T. Wanner S. Stefani S. (2014). Phenotypic and genotypic characterization of daptomycin-resistant methicillin-resistant *Staphylococcus aureus* strains: relative roles of mprF and dlt operons. *PLoS ONE* 9:e107426 10.1371/journal.pone.0107426 PMC4166420 25226591

[B45] Nawrocki K. Crispell E. Mcbride S. (2014). Antimicrobial peptide resistance mechanisms of Gram-positive bacteria. *Antibiotics* 3 461 10.3390/antibiotics3040461 PMC4239024 25419466

[B46] Nieto M. Perkins H. R. (1971). Modifications of the acyl-d-alanyl-d-alanine terminus affecting complex-formation with vancomycin. *Biochem. J.* 123 789–803. 10.1042/bj1230789 5124386 PMC1177079

[B47] Oliver J. D. (2005). The viable but nonculturable state in bacteria. *J. Microbiol.* 43 93–100. 15765062

[B48] Orman M. A. Brynildsen M. P. (2013a). Dormancy is not necessary or sufficient for bacterial persistence. *Antimicrob. Agents Chemother.* 57 3230–3239. 10.1128/AAC.00243-13 23629720 PMC3697331

[B49] Orman M. A. Brynildsen M. P. (2013b). Establishment of a method to rapidly assay bacterial persister metabolism. *Antimicrob. Agents Chemother.* 57 4398–4409. 10.1128/AAC.00372-13 23817376 PMC3754326

[B50] Paenke I. Sendhoff B. Rowe J. Fernando C. (2007). “On the adaptive disadvantage of Lamarckianism in rapidly changing environments,” in *Proceedings of the 9th European conference on Advances in Artificial Life* (Lisbon, Portugal: Springer-Verlag).

[B51] Parenti F. (1986). Structure and mechanism of action of teicoplanin. *J. Hosp. Infect.* 7 79–83. 10.1016/0195-6701(86)90011-3 2871101

[B52] Patra P. Klumpp S. (2013). Population dynamics of bacterial persistence. *PLoS ONE* 8:e62814 10.1371/journal.pone.0062814 PMC3652822 23675428

[B53] Pinchuk L. M. Degteva G. K. Samoǐlova L. N. (1983). Characteristics of the composition of higher fatty acids in methicillin-resistant and methicillin-sensitive *Staphylococci*. *Antibiotiki* 28 412–417. 6881950

[B54] Porter J. Edwards C. Pickup R. W. (1995). Rapid assessment of physiological status in *Escherichia coli* using fluorescent probes. *J. Appl. Bacteriol.* 79 399–408. 10.1111/j.1365-2672.1995.tb03154.x 7592133

[B55] Prax M. Bertram R. (2014). Metabolic aspects of bacterial persisters. *Front. Cell. Infect. Microbiol.* 4:148 10.3389/fcimb.2014.00148 PMC4205924 25374846

[B56] Rainey P. Beaumont H. Ferguson G. Gallie J. Kost C. Libby E. (2011). The evolutionary emergence of stochastic phenotype switching in bacteria. *Microb. Cell Fact.* 10:S14 10.1186/1475-2859-10-S1-S14 PMC3231921 21995592

[B57] Ramamurthy T. Ghosh A. Pazhani G. P. Shinoda S. (2014). Current perspectives on viable but non-culturable (VBNC) pathogenic bacteria. *Front. Public Health* 2:103 10.3389/fpubh.2014.00103 PMC4116801 25133139

[B58] Reynolds P. E. (1989). Structure, biochemistry and mechanism of action of glycopeptide antibiotics. *Eur. J. Clin. Microbiol. Infect. Dis.* 8 943–950. 10.1007/bf01967563 2532132

[B59] Rybak M. J. Lomaestro B. M. Rotscahfer J. C. Moellering R. C. Craig W. A. Billeter M. (2009). Vancomycin therapeutic guidelines: a summary of consensus recommendations from the Infectious Diseases Society of America, the American Society of Health-System Pharmacists, and the Society of Infectious Diseases Pharmacists. *Clin. Infect. Dis.* 49 325–327. 10.1086/600877 19569969

[B60] Sinensky M. (1974). Homeoviscous Adaptation—a homeostatic process that regulates the viscosity of membrane lipids in *Escherichia coli*. *Proc. Natl. Acad. Sci. U.S.A.* 71 522–525. 10.1073/pnas.71.2.522 4360948 PMC388039

[B61] Singh R. Ray P. Das A. Sharma M. (2009). Role of persisters and small-colony variants in antibiotic resistance of planktonic and biofilm-associated *Staphylococcus aureus*: an in vitro study. *J. Med. Microbiol.* 58 1067–1073. 10.1099/jmm.0.009720-0 19528167

[B62] Singh V. K. Hattangady D. S. Giotis E. S. Singh A. K. Chamberlain N. R. Stuart M. K. (2008). Insertional inactivation of branched-chain α-keto acid dehydrogenase in *Staphylococcus aureus* leads to decreased branched-chain membrane fatty acid content and increased susceptibility to certain stresses. *Appl. Environ. Microbiol.* 74 5882–5890. 10.1128/AEM.00882-08 18689519 PMC2565972

[B63] Stein G. E. Wells E. M. (2010). The importance of tissue penetration in achieving successful antimicrobial treatment of nosocomial pneumonia and complicated skin and soft-tissue infections caused by methicillin-resistant *Staphylococcus aureus*: vancomycin and linezolid. *Curr. Med. Res. Opin.* 26 571–588. 10.1185/03007990903512057 20055750

[B64] Wakamoto Y. Dhar N. Chait R. Schneider K. Signorino-Gelo F. Leibler S. (2013). Dynamic persistence of antibiotic-stressed mycobacteria. *Science* 339 91–95. 10.1126/science.1229858 23288538

[B65] Westwell M. S. Gerhard U. Williams D. H. (1995). Two Conformers of the glycopeptide antibiotic teicoplanin with distinct ligand binding sites. *J. Antibiot.* 48 1292–1298. 10.7164/antibiotics.48.1292 8557571

[B66] White D. C. Frerman F. E. (1968). Fatty acid composition of the complex lipids of *Staphylococcus aureus* during the formation of the membrane-bound electron transport system. *J. Bacteriol.* 95 2198–2209. 5669897 10.1128/jb.95.6.2198-2209.1968 PMC315154

[B67] Xia J. Sinelnikov I. V. Han B. Wishart D. S. (2015). MetaboAnalyst 3.0—making metabolomics more meaningful. *Nucleic Acids Res.* 43 W251–W257. 10.1093/nar/gkv380 25897128 PMC4489235

[B68] Xu H.-S. Roberts N. Singleton F. L. Attwell R. W. Grimes D. J. Colwell R. R. (1982). Survival and viability of nonculturable *Escherichia coli* and *Vibrio cholerae* in the estuarine and marine environment. *Microb. Ecol.* 8 313–323. 10.2307/4250723 24226049

[B69] Zhang Y.-M. Rock C. O. (2008). Membrane lipid homeostasis in bacteria. *Nat. Rev. Microbiol.* 6 222–233. 10.1038/nrmicro1839 18264115

